# Evolution of the Aroma Volatiles of Pear Fruits Supplemented with Fatty Acid Metabolic Precursors

**DOI:** 10.3390/molecules191220183

**Published:** 2014-12-02

**Authors:** Gaihua Qin, Shutian Tao, Huping Zhang, Wenjiang Huang, Juyou Wu, Yiliu Xu, Shaoling Zhang

**Affiliations:** 1 Department of Horticulture, Nanjing Agricultural University, Nanjing 210095, China; E-Mails: qghahstu@163.com (G.Q.); taost@njau.edu.cn (S.T.); zhanghuping@126.com (H.Z.); wenjiangh@126.com (W.H.); juyouwu@njau.edu.cn (J.W.); 2 Horticultural Research Institute, Anhui Academy of Agricultural Sciences, Hefei 230031, China; 3 Key Laboratory of Genetic Improvement and Ecophysiology of Horticultural Crop, Hefei 230031, China

**Keywords:** pear, aroma volatiles, fatty acid metabolism

## Abstract

To examine the biochemical metabolism of aroma volatiles derived from fatty acids, pear fruits were incubated *in vitro* with metabolic precursors of these compounds. Aroma volatiles, especially esters, were significantly increased, both qualitatively and quantitatively, in pear fruits fed on fatty acid metabolic precursors. Cultivars having different flavor characteristics had distinctly different aroma volatile metabolisms. More esters were formed in fruity-flavored “Nanguoli” fruits than in green-flavored “Dangshansuli” fruits fed on the same quantities of linoleic acid and linolenic acid. Hexanal and hexanol were more efficient metabolic intermediates for volatile synthesis than linoleic acid and linolenic acid. Hexyl esters were the predominant esters produced by pear fruits fed on hexanol, and their contents in “Dangshansuli” fruits were higher than in “Nanguoli” fruits. Hexyl esters and hexanoate esters were the primary esters produced in pear fruits fed on hexanal, however the content of hexyl ester in “Dangshansuli” was approximately three times that in “Nanguoli”. The higher contents of hexyl esters in “Dangshansuli” may have resulted from a higher level of hexanol derived from hexanal. In conclusion, the synthesis of aroma volatiles was largely dependent on the metabolic precursors presented.

## 1. Introduction

Fruit aromas consist of many aroma volatiles, and their formation involves several biosynthetic pathways. Fatty acid metabolism is an important metabolic pathway involved in the biosynthesis of aroma volatiles in fruits. Fatty acids liberated by lipase activity and those further metabolized by β-oxidative enzymes and/or lipoxygenase (LOX) are generally regarded as the initial precursors of straight-chain esters, alcohols, and aldehydes produced in fruits during development and maturation [1,2,3].

Transgenic modification of fatty acid metabolism in plant tissues resulted in significant changes in aroma compound profiles [4]. For fruits, higher enzymatic activities and smaller amounts of linoleic acid and linolenic acid were measured in ripe fruits compared to green fruits. Fatty acid deficiency in fruits was the primary cause of the poor aroma of fruits [1,5,6,7,8].

Studies examining aroma production in fruits feeding on short or long-chain fatty acids (FA) (up to 16 C atoms) [2,6,7] have shown that aroma production increases, even under conditions in which natural aroma volatiles production is limited by storage. Such results have demonstrated the importance of fatty acids as precursors for aroma volatile biosynthesis and the feasibility of *in vitro* aroma volatile biosynthesis modifications.

*Pyrus bretschneideri* Dangshansuli is a pear cultivar widely planted in China for its great size, rich juice and sweet, crisp flesh. The ripe fruits contain many straight-chain aldehydes and alcohols, giving them a “green” flavor note. *Pyrus ussuriensis* Nanguoli is another cultivar widely planted in the northern China for its attractive color, exquisite flesh and pleasant flavor. Esters are the important volatiles of the ripe fruits, endowing them a “fruity” flavor note [9]. The two cultivars grow well in the Liaoning Province of China and have similar bloom and maturity dates, but their aroma volatile composition and concentration are distinctly different. The majority of plant aroma volatiles originate on a quantitative and qualitative basis from saturated and unsaturated fatty acids [10]. Most aroma volatiles of fruit are biosynthetically derived from fatty acids, however the fatty acid metabolism of aroma volatiles in different cultivars have not been thoroughly examined. In this study, the aroma volatile metabolisms of “Dangshansuli” and “Nanguoli” were examined by incubating fruits with linoleic acid, linolenic acid, hexanol and hexanal to further understand aroma volatile biosynthesis and regulation.

## 2. Results and Discussion

### 2.1. Contents of Linoleic Acid and Linolenic Acid for Pear Fruits Feeding on Linoleic Acid and Linolenic Acid

**Figure 1:**
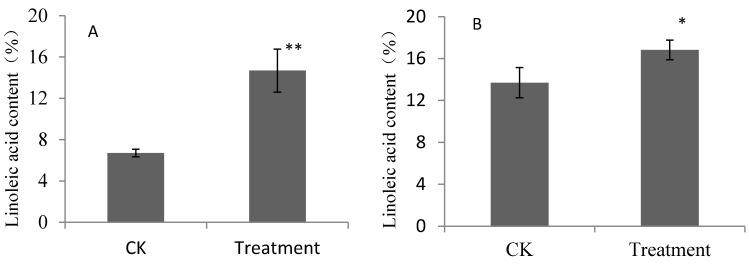
Linoleic acid contents of pear fruits feeding on linoleic acid. (**A**) *P. bretschnrideri *“Dangshansuli”; (**B**) *P. ussuriensis* “Nanguoli”; Values represented means of three replicates, bars were standard deviation from three replicates; * denoted significant difference between treatments and CK, *p* < 0.05; ** denoted significant difference between treatments and CK, *p* < 0.01.

**Figure 2:**
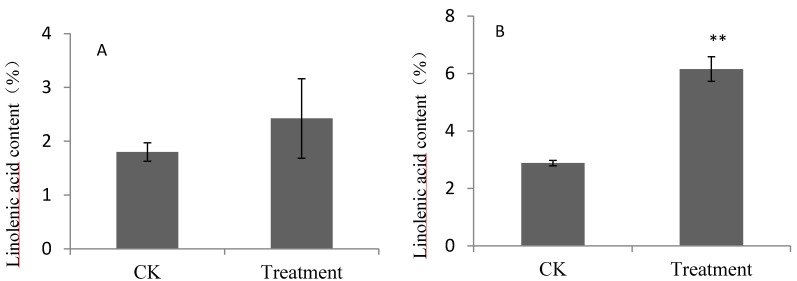
Linolenic acid contents of pear fruits feeding on linolenic acid. (**A**) *P. bretschnrideri* “Dangshansuli”; (**B**) *P. ussuriensis* “Nanguoli”; Values represented means of three replicates, bar were standard deviation; ** denoted significant difference between treatments and CK, *p* < 0.01.

*In vitro* cultures are useful for examining the biosynthetic pathways of aroma volatiles in fruits [11,12,13]. The incubation of pear fruits with linoleic acid and linolenic acid showed that these compounds were efficiently absorbed by pear fruits (Figure 1 and Figure 2) and used for the synthesis of aroma volatiles. However, different absorbabilities were observed for the two pear cultivars. 

Linoleic acid content increased by 7.98% and 3.13% for “Dangshansuli” and “Nanguoli” fruits fed on linoleic acid, respectively. The content of linoleic acid in “Dangshansuli” fruits fed on linoleic acid was 2.19-fold that of the control, while it was 1.23-fold that of the control for “Nanguoli” fruits.

The content of linolenic acid increased for pear fruits fed on linolenic acid, however the increase was not significant for “Dangshansuli” fruits. Linolenic acid content increased by 0.62% and 3.27% for “Dangshansuli” and “Nanguoli” fruits fed on linolenic acid, respectively. These results show that “Dangshansuli” fruits absorbed linoleic acid much more effectively than linolenic acid, while “Nanguoli” fruits had similar absorption capacities for linolenic acid and linoleic acid.

### 2.2. Composition of Aroma Volatiles Derived from Fatty Acid Metabolic Pathway for Pear Fruits Feeding on Linoleic Acid and Linolenic Acid

The absorbed linoleic acid and linolenic acid were used for the synthesis of straight-chain esters, alcohols and aldehydes. Esters were the class of volatile with the greatest increases in pear fruits. Methyl acetate, methyl hexanoate, methyl 2-hexenoate, ethyl hex-3-enoate, hexyl acetate, and methyl octanoate were all increased in “Dangshansuli” fruits fed on linoleic acid. The esters increased in “Nanguoli” fruits fed on linoleic acid were ethyl acetate, ethyl butanoate, butyl acetate, methyl hexanoate, and some long-chain ester compounds (Table 1). The total quantity of esters increased to 409.4 ng·g^−1^·FW and 1104.4 ng·g^−1^ FW for “Dangshansuli” and “Nanguoli” fruits fed on linoleic acid, respectively, representing 2.07-fold and 1.66-fold increases, respectively, over control levels.

Esters were also increased in pear fruits fed on linolenic acid. Methyl acetate, methyl hexanoate, and the unsaturated esters methyl 2-hexenoate, ethyl hex-3-enoate, (*Z*)-hexen-2-ol acetate, were significantly increased in fruits fed on linolenic acid. More than 10 aroma volatiles, including several long-chain esters were synthesized in pear fruits fed on linolenic acid. The total quantity of esters increased to 285.1 ng·g^−1^·FW and 1,163.4 ng·g^−1^ FW for “Dangshanli” and “Nanguoli” fruits fed on linolenic acid, respectively, representing 1.44-fold and 1.75-fold increases over control fruits. The increases in esters corresponded to the increases of linoleic acid and linolenic acid (Figure 1 and Figure 2). The relationships between volatile esters and linoleic acid and linolenic acid, induced by increases in linoleic acid and linolenic acid, or measures promoting their formation, could enhance the volatile esters biosynthesis in fruits.

Volatile esters are formed by esterification of alcohols and acyl from acyl-CoAs in a reaction catalyzed by alcohol acyltransferase (AAT) [14,15]. The concentration of substrates, including alcohols, acyl-CoAs and the precursors of these compounds in fruits, may be a limiting factor for ester production. As demonstrated in the present study, addition of linoleic acid and linolenic acid, which were precursors of many straight-chain alcohols and aldehydes, increased ester production in pear fruits. Previous experiments in apples have shown that off-flavor fruits, such as those harvested prior to reaching maturity or those stored for an extend period of time in low O_2_ and/or high CO_2_ environments, immediately produced high amounts of aroma volatiles after feeding on various volatile precursors [5]. These results validated the hypothesis that enhancing levels of linoleic acid and/or linolenic acid in fruits could increase the ester content.

As intermediate of fatty acid metabolism, the direct precursors of ester formation are also affected by the key enzymes catalyzing their production. The activities of these key enzymes may be affected by cultivar, maturity stage, and environmental conditions, either on or off of the plant [16,17,18,19,20,21]. The greater increase of esters in “Nanguoli” fruits than in “Dangshansuli” fruits fed on the same quantities of linoleic acid and linolenic acid may have been caused by differing enzyme activity in the cultivars. Additionally, (*Z*)-2-hexenyl acetate was detected only in “Dangshansuli” fruits fed on linolenic acid, ethyl (*Z*)-4-octenoate was detected only in “Nanguolii” fruits fed on linolenic acid, methyl tetradecanoate was the only ester detected in “Dangshansuli” and “Nanguolii” fruits fed on linolenic acid. The result may due to the specific recognition of key enzymes.

**Table 1:** Contents (ng·g^−1^·FW) of aroma volatiles derived from fatty acid metabolism for pear fruits feeding on linoleic acid and linolenic acid; ^(a)^ -, not detected; ^(b)^ Values represented means ± SD of three replicates; ^(c)^ Capitals denoted significant difference in a line for one cultivar at the level of 0.01. Lowercase letters denoted significant differences in a line for one cultivar at the level of 0.05, according to Duncan’s test.

Kinds	Aroma Volatiles	Dangshansuli			Nanguoli		
		CK	Linoleic Acid	Linolenic Acid	CK	Linoleic Acid	Linolenic Acid
Esters	Methyl acetate	- ^(a)^	15.7 ± 2.8 ^(b)^	14.6 ± 4.9	5.7 ± 3.1	17.6 ± 8.9	10.1 ± 3.2
	Ethyl acetate	45.9 ± 3.8	50.6 ± 7.1	55.6 ± 6.3	185.4 ± 27.7	324.8 ± 69.6	263.3 ± 115.6
	Ethyl propanoate	-	-	-	4.4 ± 1.1	6.8 ± 1.8	4.4 ± 1.0
	Methyl butanoate	0.9 ± 0.1	1.8 ± 0.0	0.7 ± 0.1	7.4 ± 1.3	31.5 ± 18.2	11.1 ± 2.1
	Methyl 2-butenoate	-	0.1 ± 0.1	0.2 ± 0.0	-	-	-
	Ethyl butanoate	64.3 ± 8.1	18.7 ± 3.7	8.8 ± 5.4	39.6 ± 13.1	132.1 ± 2.1	132.8 ± 11.4
	Butyl acetate	-	-	-	-	33.1 ± 17.2	24.2 ± 6.3
	Ethyl 2-butenoate	-	-	-	6.2 ± 2.0	7.0 ± 4.2	4.7 ± 2.0
	Ethyl pentanoate	0.2 ± 0.0	-	-	0.8 ± 0.8	0.2 ± 0.1	2.7 ± 2.4
	Pentyl acetate	1.3 ± 0.1	7.0 ± 0.8	-	1.5 ± 0.5	1.9 ± 0.8	1.3 ± 0.9
	Methyl hexanoate	1.3 ± 1.0	54.5 ± 3.9	31.4 ± 12.8	33.6 ± 8.5	231.4 ± 10.7	130.0 ± 3.3
	Methyl 2-hexenoate	0.5 ± 0.2	6.8 ± 0.9	6.7 ± 3.0	0.3 ± 0.4	3.0 ± 1.8	1.5 ± 1.1
	Ethyl Hexanoate	39.2 ± 5.6	13.1 ± 11.6	17.3 ± 10.2	187.2 ± 37.9	162.7 ± 15.1	391.2 ± 10.0
	Ethyl Hex-3-enoate	-	21.2 ± 5.5	43.7 ± 12.1	-	-	-
	Hexyl acetate	39.3 ± 14.7	165.7 ± 9.4	26.6 ± 1.5	168.5 ± 28.6	118.7 ± 6.7	136.2 ± 11.3
	(*Z*)-2-Hexenyl acetate	-	-	48.2 ± 1.0	-	-	-
	Methyl heptanoate	-	8.1 ± 1.1	3.5 ± 0.7	-	-	-
	Ethyl 2-hexenoate	0.2 ± 0.0	0.2 ± 0.1	0.3 ± 0.1	6.7 ± 1.9	10.3±6.5	10.8 ± 0.1
	Ethyl heptanoate	1.5 ± 0.5	-	-	-	-	-
	Heptyl acetate	0.2 ± 0.1	0.8 ± 0.5	-	0.2 ± 0.0	-	1.8 ± 0.2
	3-Hepten-1-ol,1-acetate	-	0.5 ± 0.3	-	-	-	-
	Methyl octanoate	-	28.8 ± 2.6	11.3 ± 1.8	-	9.6 ± 1.7	5.6 ± 0.6
	Methyl (*E*)-2-octenoate	-	2.6 ± 0.5	4.9 ± 1.2	-	1.5 ± 0.1	-
	Ethyl (*Z*)-4-octenoate	-	-	-	-	-	0.3 ± 0.1
	Hexyl butanoate	-	-	-	0.3 ± 0.1	-	-
	Ethyl octanoate	3.1 ± 0.6	0.6 ± 0.0	-	2.2 ± 1.1	0.9 ± 0.2	1.8 ± 0.2
	Methyl nonanoate	-	0.3 ± 0.1	0.4±0.0	-	-	-
	Ethyl (*E*)-2-octenoate	-	-	-	1.7 ± 0.8	1.4 ± 0.1	3.9 ± 0.1
	Methyl 4-decenoate	-	2.2 ± 0.3	1.8 ± 0.8	-	1.0 ± 0.2	-
	Methyl decanoate	-	1.0 ± 0.0	1.3 ± 0.4	-	-	-
	Hexyl hexanoate	-	0.5 ± 0.1	0.4 ± 0.1	-	-	-
	Methyl (*E,Z*)-2,4-decadienoate	-	5.4 ± 0.9	3.6 ± 1.0	2.8 ± 1.5	3.4 ± 0.2	8.5 ± 1.3
	Ethyl (*E,Z*)-2,4-decadienoate	-	0.9 ± 0.2	1.2 ± 0.4	9.6 ± 4.1	5.5 ± 1.2	19.0 ± 1.2
	Methyl dodecanoate	-	0.6 ± 0.1	0.8 ± 0.1	-	-	1.2 ± 0.3
	Methyl tetradecanoate	-	-	0.1 ± 0.0	-	-	0.5 ± 0.1
	Methyl hexadecanoate	-	1.8 ± 0.7	1.7 ± 0.2	-	-	-
	Subtotal	197.9 ^cC,^^(c)^	409.4 ^aA^	285.1 ^bB^	664.1 ^bB^	1104.4 ^aA^	1163.4 ^aA^
Aldehydes	Hexanal	16.7 ± 2.1	9.2 ± 1.6	3.7 ± 4.0	131.7 ± 23.1	44.1 ± 3.1	4.3 ± 0.6
	2-Hexenal	8.1 ± 2.6	2.6 ± 0.1	9.0 ± 7.0	-	-	-
	Nonanal	2.2 ± 0.4	0.5 ± 0.1	1.6 ± 0.4	-	-	-
	Decanal	0.9 ± 0.1	-	-	-	-	-
	Subtotal	27.9 ^aA^	12.3 ^bB^	14.3 ^bAB^	131.7 ^aA^	44.1 ^bB^	4.3 ^cC^
Alcohols	Ethanol	5.5 ± 1.7	3.1 ± 0.9	5.0 ± 2.0	7.8 ± 2.3	14.4 ± 0.1	18.1 ± 5.5
	(*E*)-2-Hexen-1-ol	0.2 ± 0.1	2.4 ± 0.2	10.2 ± 1.7	-	2.6 ± 0.2	1.7 ± 0.2
	1-Hexanol	1.9 ± 1.3	86.9 ±1.4	23.6 ± 3.3	33.3 ± 3.3	139.5 ± 1.2	4.9 ± 3.2
	1-Heptanol	-	3.2 ± 1.6	-	-	-	-
	Octen-2-ol	-	4.4 ± 0.7	-	-	-	-
	1-Octanol	0.3 ± 0.0	1.9 ± 0.5	0.5 ± 0.1	0.7 ± 0.2	0.8 ± 0.1	1.2 ± 0.1
	(*E*)-2-Octen-1-ol	-	1.7 ± 0.0	-	-	-	-
	Subtotal	7.9 ^cC^	103.6 ^aA^	39.3 ^bB^	41.9 ^bB^	157.3 ^aA^	25.9 ^cB^

The different characters of the key enzymes and the different content of linoleic acid and linolenic acid maybe explain the varying volatile ester-forming capacities in different pear cultivars. The enhancement of precursor contents may be a more feasible method of increasing volatile esters in pear fruits for a cultivar. The content of alcohols also changed significantly. The main alcohols increased in “Dangshansuli” fruits were (*E*)-2-hexen-1-ol, 1-hexanol, 1-heptanol, 1-octanol and octen-2-ol, while 1-hexanol and 1-octanol increased in “Nanguoli” fruits. The total quantity of alcohol was increased for pear fruits fed on linoleic acid, while the increase was greater for “Dangshansuli” than “Nanguoli” fruits. The total quantity of alcohol was increased in “Dangshansuli” fruits fed on linolenic acid, but decreased in “Nanguoli” fruits given this compound. The contents of aldehydes were decreased in pear fruits fed on linoleic acid and linolenic acid.

### 2.3. Contents of Hexanal and Hexanol for Pear Fruits Fed on Hexanal and Hexanol

The contents of hexanol and hexanal were significantly increased in pear fruits fed on hexanol and hexanal (Table 2). This result indicated hexanol and hexanal could be efficiently absorbed by pear fruits. The content of hexanol in “Nanguoli” fruits was similar to that in “Dangshansuli” fruits when fed the same quantity of hexanal, while the content of hexanol in “Nanguoli” fruits was higher than that in “Dangshansuli” fruits when fed the same quantity of hexanol. The content of hexanol was significant greater than that of hexanal in pear fruits fed on hexanal or hexanol.

**Table 2:** Hexanol and hexanal contents (ng·g^−^1·FW) of pear fruits fed on hexanol and hexanal; ^(a)^ -, not detected. ^(b)^ Values represented means ± SD of three replicates. ^(c)^ ** denoted significant differences between treatments and CK at the level of 0.01, according to Duncan’s test.

Treatments	Dangshansuli		Nanguoli	
	Hexanal	Hexanol	Hexanal	Hexanol
CK	16.7 ± 2.1 ^(b)^	1.9 ± 1.3	- ^(a)^	37.9 ± 10.0
Hexanal	19.0 ± 0.2	1909.3 ± 39.8 **^, ^^(c)^	19.7 ± 10.3 **	1822.4 ± 748.1 **
Hexanol	20.3 ± 0.3	2193.9 ± 47.8 **	11.0 ± 15.6 **	3024.8 ± 537.3 **

### 2.4. Hexyl Ester and/or Hexanoate Ester Content in Pear Fruits Fed on Hexanal and Hexanol

Hexanol and hexanal are important intermediates of the fatty acid metabolic pathway. Some of the hexanol and hexanal in fruits shuttle across cell membranes and alter fruit aroma volatiles, while the others are used as precursors for ester formation. Hexanol can be directly used to synthesis volatile esters catalyzed by AAT. Hexanal, as an intermediate metabolite, is another important substrate of volatile esters biosynthesis. As the reduced form of hexanal, hexanol can be used in the syntheses of hexyl esters. Similarly, as the oxidized form of hexanal, hexanoate can be used to form hexanoate esters.

The content of hexyl ester and hexanoate ester were increased in pear fruits feeding on hexanal and hexanol due to the increase in precursor substrates of ester biosynthesis (Table 3). Hexyl esters content, especially those of hexyl acetate, hexyl butanoate, and hexyl hexanoate, were significantly increased in pear fruits fed on hexanol. The total quantity of hexyl ester reached 1524.2 ng·g^−1^ FW and 1127.4 ng·g^−1^ FW for “Dangshansuli” and “Nanguoli” fruits fed on hexanol, respectively. These values represented increases of 1485.1 ng·g^−1^ FW and 958.6 ng·g^−1^ FW, separately, over the control fruits. The greater increases of hexyl ester content in “Dangshansuli” fruits than “Nanguoli” fruits fed on the same quantity of hexanol demonstrated that “Dangshansuli” fruits were capable of forming more hexyl ester than “Nanguoli” fruits when given sufficient hexanol.

**Table 3:** Hexyl and hexanoate esters contents (ng·g^−1^·FW) of pear fruits feeding on hexanol and hexanal ^(a)^ -, not detected; ^(b)^ Values represented means of three replicates; ^(c)^ Capitals denoted significant differences in a line for one cultivar at the level of 0.01. Lowercase denoted significant differences in lines for one cultivar at the level of 0.05.

Aroma Volatiles	Dangshansuli			Nanguoli		
	CK	Hexanal	Hexanol	CK	Hexanal	Hexanol
Methyl hexanoate	1.3 ± 1.0 bAB ^(b)^	5.4 ± 4.2 aA	- ^(a)^	33.6 ± 8.5 bB	81.0 ± 24.6 aA	25.8 ± 1.82 bB
Ethyl hexanoate	39.2 ± 5.6 cC	526.0 ± 22.9 bB	114.6 ± 4.8 aA	187.2 ± 37.9 bB	621.5 ± 226.4 aA	160.5 ± 76.0 bB
Hexyl acetate	39.3 ± 14.7 bB	1370.4 ± 162.9 aA	1350.2 ± 284.8 aA	168.5 ± 28.6 cB	594.3 ± 303.8 bAB	1006.1 ± 71.1 aA
Hexyl butanoate	-	72.4 ± 4.3 aA	50.0 ± 24.1 bA	0.3 ± 0.1 cC	35.0 ± 10.9 bB	79.7 ± 19.0 aA
Hexyl hexanoate	-	272.6 ± 79.9 aA	120.7 ± 38.2 bB	-	137.7 ± 42.9 aA	41.6 ± 58.9 bB
Hexyl benzoate	-	0.5 ± 0.1 bB	1.3 ± 0.4 aA	-	-	-
Hexyl octanoate	-	0.9 ± 0.2 bB	2.2 ± 1.0 aA	-	-	-
Total contents	79.8 ± 21.2 cC ^(c)^	2248.2 ± 274.6 aA	1639.0 ± 353.2 bB	389.60 ± 75.1 bB	1469.5 ± 608.6 aA	1313.8 ± 153.9 aA

The dominant esters for pear fruits fed on hexanal were hexyl acetate and ethyl hexanoate, followed by hexyl hexanoate. The content of hexyl acetate in “Dangshansuli’ fruits fed on hexanal was 2.6-fold that of ethyl hexanoate, while the contents of ethyl hexanoate and hexyl acetate were similar in “Nanguoli” fruits fed on hexanal. The total quantity of hexanoate esters was 804.0 ng·g^−1^·FW and 840.2 ng·g^−1^·FW, respectively, for “Dangshansuli” and “Nanguoli” fruits fed on the same quantity of hexanal, representing increases of 759.5 ng·g^−1^·FW and 619.4 ng·g^−1^·FW. The content of hexyl esters were 1716.8 ng·g^−1^·FW and 767.0 ng·g^−1^·FW for “Dangshansuli” and “Nanguoli” fruits fed on the same quantity of hexanal, representing increases of 1677.5 ng·g^−1^·FW and 598.2 ng·g^−1^·FW, respectively. Thus, we concluded that the greater amount of hexanal absorbed by “Dangshansuli” fruits was reduced to hexanol for the synthesis hexyl esters, but not oxidized to hexanoate for the synthesis hexanoate esters, while similar quantities of hexanol and hexanoate were formed to generate the corresponding esters in “Nanguoli” fruits fed on hexanal.

The increase of hexanol in “Nanguoli” fruits was higher than that in “Dangshansuli” fruits when they were fed on the same quantity of hexanol (Table 2). However, the content of the corresponding hexyl esters in “Nanguoli” fruits was two-thirds that in “Dangshansuli” fruits (Table 3). The comparatively low hexyl esters content in “Nanguoli” fruit fed on hexanol have been due to the greater released from fruits or unknown differences in the capacity for ester formation between cultivars remained unknown. If it was the metabolic difference, the difference may be due to the low levels of acetate or decreased enzyme activity for ester synthesis, as well as feedback inhibition. Feedback inhibition of volatile aroma biosynthesis has been observed in *Antirrhinum majus* [22]. Whether the low hexyl acetate formation for “Nanguoli” fruits fed on hexanol resulted from decreased AAT activity *in vitro* or feedback inhibition requires further analysis. 

Different increases of hexyl esters were observed in different pear fruits feeding on same quantity of hexanal. Higher levels of hexyl ester in “Dangshansuli” fruits showed that more hexanol was formed in “Dangshansuli” fruits after feeding on hexanal. The activity levels of reductase/oxidase were responsible for the aroma volatiles synthesis. In “Dangshansuli” fruits, more hexanal was reduced to hexanol catalyzed by ADH reductase, so the activity of ADH reductase may not the limiting factor in volatile ester biosynthesis for these fruits. The low levels of hexyl esters in “Nanguoli” fruits fed on hexanal indicated the limitation of ADH reductase in catalyzing hexanal to hexanol. That ADH reductase was a limiting factor for aroma biosynthesis in “Nanguoli” fruits remains a hypothesis and requires further study.

The higher ester content in pear fruits fed on hexanal and hexanol than fruits fed on linoleic acid and linolenic acid showed that the former two compounds more efficiently enhanced volatile biosynthesis, which may have been due to comparatively better uptake and metabolism of these compounds. A similar phenomenon has been observed in branched chain flavor volatile synthesis by amino acid metabolism pathway. The application of amino acid to fruit pericarp segments did not stimulate synthesis of the corresponding volatile, however application of the corresponding α-keto acids resulted in much higher volatile production [23]. Therefore, the content of hexanal and hexanol or the formation rate are likely more important in the production of aroma volatiles than linoleic acid and linolenic acid. Metabolic intermediates have been indicated as more important than precursors in aroma compound synthesis for other fruits [13]. Due to the importance of the precursors hexanal and hexanol, the initial steps (including those catalyzed by the key enzymes LOX and HPL) of hexanal and hexanol formation are critical for ester biosynthesis in pear fruits. In plants, hexanal is synthesized through the conversion of linolenic acid or linoleic acid via the enzymes LOX and HPL. Synthesis of green note aroma compounds through the biotransformation of fatty acids using yeast cells co-expressing lipoxygenase and hydroperoxide lyase indicates that modification of LOX and HPL activity may serve as a promising approach for increasing efficiency of green note aldehyde synthesis [24]. Several studies have demonstrated that LOX plays a key role in volatile ester production [16,17,18]. Accordingly, further studies of HPL and ADH, which catalyze aldehydes to their corresponding alcohols in pear fruits, should be taken.

## 3. Experimental Section

### 3.1. Plant Materials

In this study, “*Pyrus bretschnrideri*” “Dangshansuli’’ and “*Pyrus ussuriensis*” “Nanguoli’’ fruits of the same age and grown under the similar cultivation measures, including irrigation and fertilization, were acquired from the Liaoning Institute of Pomology (Xiongyue, China). Twenty ripe fruits of uniform size and color and free of visible defects or decay were harvested for each cultivar and stored under refrigeration.

### 3.2. Precursor Substrates and Incubation Method

Each precursor standard (linoleic acid, linolenic acid, hexanal and hexanol) was dissolved in 0.4 mM sorbitol, and several drops of Tween-20 were added to promote dissolution. Individual 1 mM solutions of linoleic acid, linolenic acid, hexanal and hexanol solution were obtained individually for incubation in this study. Linoleic acid, linolenic acid, hexanal and hexanol standards were all of analytical grade and purchased from Sigma-Aldrich (St. Louis, MO, USA). Sorbitol was chemically pure and purchased from Sinopharm Chemical Reagent Co., Ltd (Shanghai, China). Fruits were removed from cold storage and incubated at room temperature for 24 h, then cut into discs with a diameter of 1 cm and a thickness of 0.2 cm. Incubation of fruits with the precursor substrates was conducted as described by Zhang [25]. Fruit discs were divided into five portions. The first portion was placed in 0.4 mM sorbitol and used for a control (CK), the other portions were placed into the different substrate solutions and incubated for 8 h at 35 °C while shaking at 60 r·min^−1^. After incubation, the discs were blotted using a filter paper, frozen in liquid nitrogen, and stored at −80 °C until analysis.

### 3.3. Extraction of Polar Lipids

Fruit tissue (2 g) was ground in an ice bath and chloroform/methanol (6 mL, 1:2, v/v) was added in the flesh. The tissue was extracted for 20 min at 4 °C, then centrifuged at 3000× *g* for 7 min. The resulting extract was again subjected to extraction with chloroform/methanol (3 mL, 1:2, v/v) and chloroform (4 mL) was then used to purge. All of the resultant extracts were combined, 0.76% NaCl (w/v) (20 mL) was added, and the samples were shaken on a shaking table (WZS-200A, Selon Scientific Instrument Co., Ltd., Shanghai, China) for 15 min. The aqueous layer was removed and the residue was vaporized using a rotary evaporator (RE-3000, Shanghai Yarong Instrument Factory, Shanghai, China) to obtain the polar lipids. The polar lipids were dissolved in petroleum ether (90–120 °C boiling point range) with saturated methanol (6 mL), and methanol saturated with petroleum ether (6 mL) was then used to purify them. This purification process was repeated three times. Finally, the pure polar lipid was dissolved in methanol (2 mL, gas chromatography (GC) grade) for esterification.

The polar lipids were then methyl esterified. The polar lipid was redissolved in 0.4 M KOH in methanol (2 mL) and benzene-petroleum ether (30–60 °C boiling point range, 2 mL, 1:1, v/v), vibrated, and then allowed to rest for 15 min. Distilled water (16 mL) was added to the vials, and the upper layer of the extracts was withdrawn and vaporized using a vacuum machine, and then redissolved in hexane (1 mL) for gas chromatography analysis.

### 3.4. Analysis of Fatty Acid by GC

Fatty acids were analyzed using an Agilent 7820 gas chromatograph (Santa Clara, CA, USA) equipped with flame ionization detector (FID) detector and DB-WAX column, (30 cm × 0.25 mm × 0.25 µm, Agilent). A volume of 5 µL of extract was injected for analyses. Helium was used as the carrier gas (1.0 mL·min^−1^) in constant flow mode, with a split ratio of 1:10. Injector and detector temperatures were set at 250 °C and 280 °C, respectively. For the temperature gradient, the oven was set at an initial temperature of 50 °C for 1 min, which was increased to 200 °C at a rate of 25 °C·min^−1^, then raised to 230 °C at a rate of 3 °C·min^−1^, and held at 230 °C for 18 min.

### 3.5. Identification and Quantification of Fatty Acids

Fatty acids were identified using fatty acid methyl ester mixture (FAME, C4-C24, Sigma), and quantified using normalization methods. Fatty acid content is denoted as a percent.

### 3.6. Extraction and Concentration of Aroma Volatile of Pear Fruits

Head space solid-phase microextraction (HS-SPME) was used to extract and concentrate the aroma volatiles as described by Qin [26]. The volatiles were fused with silica fiber coated with 65 µm of polydimethylsiloxane-divinylbenzene (65 µm of PDMS/DVB; Supelco Co., Bellefonte, PA, USA). Next, fruit tissue (10.0 g) was placed into a 20 mL screw-cap vial containing NaCl (3.6 g, to facilitate the release of volatile compounds) and 0.04 g·mL^−1^ 3-nonanone (50 μL, used as an internal standard). The SPME fiber was exposed to the head space of the sample for 30 min to adsorb the analyte, then introduced into the heated injector port of the chromatography apparatus for desorption at 250 °C for 5 min in splitless mode.

### 3.7. GC-Mass Spectrometry (MS) Analysis of Aroma Volatiles

Volatile constituents were analyzed using an Agilent 5973B mass selective detector coupled to an Agilent 7890A gas chromatograph equipped with a 30 m × 0.25 mm × 1.0 mm HP-5 MS (5% phenyl-polymethylsiloxane) capillary column. A constant column flow of 1.0 mL/min helium was used as carrier gas. The injector and detector temperatures were 250 °C and 280 °C, respectively. The oven temperature program was 35 °C for 8 min, an increase of 2 °C/min to 140 °C, 140 °C for 2 min, and an increase of 10 °C/min to 260 °C, which was held for 5 min. Mass spectra were recorded at 70 eV in electron impact (EI) ionization mode. The temperatures of the quadrupole mass detector and ion source were 150 °C and 230 °C, respectively. The temperature of the transfer line was 280 °C. Mass spectra were scanned in the *m*/*z* range 33–350 amu at intervals of 1 s.

### 3.8. Identification and Quantification of Aroma Volatiles

Tentative identification of aroma volatiles was conducted by comparing the mass spectra of the samples with the data system library (NIST 98). Whenever possible, MS identification was confirmed using authentic references for ethyl acetate, butyl acetate, ethyl hexanoate, hexyl acetate, ethyl octanoate, ethyl (*E,Z*)-2,4-decadienoate, 1-hexanol and hexanal were used in the experiment. Quantification was conducted using the internal standard method, where the concentration of each aroma volatile was normalized to that of 3-nonanone.

### 3.9. Statistical Analysis

The SPSS 16.0 statistical software package (IBM, Armonk, NY, USA) and Excel were used for statistical analysis. All data were generated from triplicate experiments and reported as the average of three replicates. Analysis of variance (ANOVA) was calculated and the significance of differences was detected by using Duncan’s test or Tukey’s test.

## 4. Conclusions

Aroma volatiles, particularly volatile esters, were significantly increased both qualitatively and quantitatively in pear fruits fed on fatty acid metabolic precursors. The quantity of aroma volatiles was largely dependent on the quantity of precursors presented. Hexanal and hexanol were more efficient for promoting volatile synthesis than linoleic acid and linolenic acid. More volatile esters were synthesized by “Dangshansuli” fruits than by “Nanguoli” fruits fed on the same quantity of hexanal and hexanol, suggesting that the green-flavored “Dangshansuli” fruits had less endogenous hexanal and hexanol to form volatile esters. Hexanal and hexanol contents and their formation rates may be rate-limiting factors for volatile ester production in pear fruits. 
